# Revitalizing the Smile: A Case Report on the Modified Apexification Procedure for Discolored Non-Vital Immature Teeth With Periapical Lesions

**DOI:** 10.7759/cureus.71140

**Published:** 2024-10-09

**Authors:** Yash Sinha, Twinkle Agarwal, Ishika Chatterjee, Prasanti Pradhan

**Affiliations:** 1 Department of Conservative Dentistry and Endodontics, Kalinga Institute of Dental Sciences, Kalinga Institute of Industrial Technology (KIIT) Deemed to be University, Bhubaneswar, IND; 2 Department of Conservative Dentistry and Endodontics, Institute of Dental Sciences, Siksha 'O' Anusandhan (SOA) Deemed to be University, Bhubaneswar, IND

**Keywords:** mineral trioxide aggregates, mta apexification, open apex, regenerative endodontic procedure, walking bleach

## Abstract

Apexification is the process of forming mineralized tissue at the apical portion of a tooth with an incompletely formed root. Although various materials and techniques for the endodontic treatment of such teeth have been employed for some time, selecting the appropriate material remains challenging due to limited literature. This report presents a case that evaluates the efficacy of a modified apexification procedure using mineral trioxide aggregate (MTA), followed by a walking-bleach technique to address discoloration resulting from trauma to anterior teeth. The patient was followed for 12 months after the procedure, during which we observed positive clinical and radiographic changes. The lesion size decreased, and root formation was completed, accompanied by a significant improvement in tooth shade. Based on our findings, we conclude that modified apexification followed by non-vital bleaching can be an effective treatment option for discolored trauma-affected teeth with incomplete root formation.

## Introduction

Treating teeth compromised by infection or trauma prior to full root development presents significant challenges. Successful endodontic treatment depends on the closure of the apical foramen with calcified tissue, a critical step in the maturation process [1]. Apexification, a procedure designed to induce the formation of mineralized tissue in the apical region of an immature tooth, is essential in such cases. Over time, various techniques have emerged to facilitate this process [2]. Early approaches utilizing calcium hydroxide have evolved into contemporary methods incorporating mineral trioxide aggregate (MTA), reflecting advancements in endodontic therapy [3].

Calcium hydroxide apexification requires multiple visits and prolonged follow-up, which can lead to patient noncompliance [4]. This lack of compliance may result in reinfection or fracture of temporary restorations [5]. Although regenerative endodontic procedures (REPs) demonstrate continuous root development in necrotic and immature teeth [6], their outcomes can be unpredictable, highlighting the need for more definitive treatments [7,8].

MTA, known for its excellent biocompatibility and sealing ability, stimulates the development of new mineralized tissue [9] and is often used in single-sitting apexification [10]. While the method of apical closure using MTA and pulp revascularization shows promise [11], the presence of wide-open apices may lead to the apical extrusion of materials, especially for calcium silicate-based cement and endodontic sealers with lengthy setting times. This extrusion could hinder the healing of peri-apical lesions [12]. Additionally, the chemical and thermal gutta-percha technique is not effective for cases with open apices [3].

Hemostatic collagen membranes have been found to prevent the apical extrusion of substances [3,13], but there remains a need for an autogenous material. Platelet-rich fibrin (PRF), composed of platelets and growth factors [14], serves as an effective apical barrier for MTA and Biodentine placement [15,16]. Both leukocyte-PRF and advanced-PRF have demonstrated favorable results [2,17].

## Case presentation

**Figure 1:**
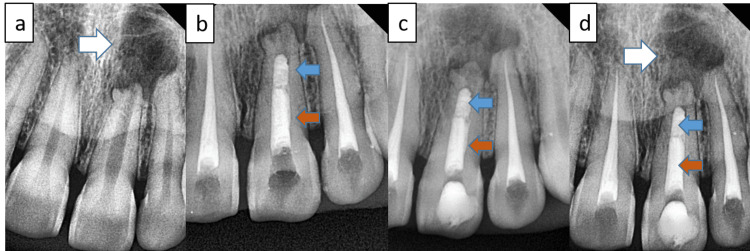
(a) Preoperative radiograph showing the open apex in relation to tooth 21. (b) Radiograph following the modified apexification procedure for tooth 21. (c) Immediate follow-up radiograph. (d) Radiograph taken 12 months post-procedure. The white arrow indicates the preoperative and postoperative radiographs demonstrating a decrease in the size of the periapical lesion. The blue arrow highlights the placement of MTA, while the orange arrow points to the thermoplastic gutta-percha. MTA, mineral trioxide aggregate

A 25-year-old female presented with mild-to-moderate continuous pain in the upper front tooth region for two weeks. Clinical examination revealed tenderness to percussion in teeth 11, 21, and 22, along with mild discoloration of tooth 21. A radiographic examination of tooth 21 showed an immature open apex and periapical radiolucency (Figure 1a).

The patient was diagnosed with a symptomatic periapical abscess involving teeth 11, 21, and 22. Given her dental trauma history and the immature root apex, a modified apexification procedure with PRF membrane reinforcement was chosen for tooth 21, in conjunction with conventional root canal treatment for teeth 11 and 22.

An Endo-Z bur (Dentsply Maillefer, Ballaigues, Switzerland) was used to create an access opening under rubber dam isolation and local anesthesia with 2% lidocaine containing 1:100,000 epinephrine. A No. 15 K-file was employed to achieve apical patency, with the working length measured using an apex locator (Root ZX Mini, J. Morita, Saitama, Japan) and confirmed radiographically. Cleaning and shaping were accomplished with hand K-files (Mani, New Delhi, India) and ProTaper Universal rotary files (Dentsply Maillefer). The canals were irrigated with 3% sodium hypochlorite (Hyposol; Prevest DenPro, Jammu, India), 17% EDTA (Neoedta; Orikam, Gurgaon, India), and saline, followed by drying with sterile paper points.

After two weeks of calcium hydroxide medication (RC Cal; Prime Dental, Thane, India), the root canal was disinfected using 3% sodium hypochlorite, and a PRF membrane was prepared from the patient’s venous blood. A 10-milliliter blood sample was drawn from the patient’s antecubital vein and centrifuged at 1,300 revolutions per minute for eight minutes, producing a maximum relative centrifugal force (RCF-max) of 208 times gravity. Following centrifugation, the platelet-enriched fibrin matrix was carefully extracted and allowed to form a membrane on a flat surface for two minutes. The membrane was then divided into 3-mm squares, which were gently compacted into the apical region using a specialized instrument. Additional segments of the membrane were incrementally condensed into the periapical area using a condenser, ultimately forming a stable barrier 1-2 mm thick near the root apex.

Following the manufacturer’s guidelines, MTA Angelus (Angelus, Londrina, Brazil) was mixed and delivered apically using a carrier. The material was packed against the PRF barrier with a plugger, ensuring 3-4 mm of MTA placement. Radiographic verification confirmed proper placement and absence of extrusion. The remaining canal space was filled with thermoplastic gutta-percha (EQV, Meta Biomed, Cheongju-si, South Korea) and AH Plus sealer (Dentsply Maillefer) (Figure 1b, 1c).

**Figure 2:**
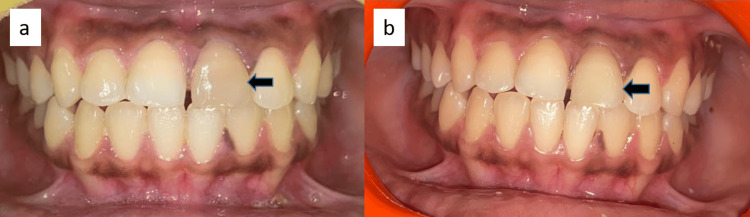
(a) Preoperative photograph showing discoloration associated with tooth 21. (b) Postoperative photograph following bleaching treatment for tooth 21. The black arrow indicates the tooth of concern.

One week later, the patient returned for in-office bleaching. To prepare for the procedure, 1-2 mm of gutta-percha was removed below the cementoenamel junction, and a layer of glass ionomer cement was applied over the remaining gutta-percha. A 35% hydrogen peroxide gel (Opalescence™ Endo, Ultradent Products, Inc., South Jordan, United States) was placed inside the pulp chamber and compressed with a damp cotton pellet. The access cavity was sealed with Cavit. Due to minimal color improvement, the procedure was repeated after one week. Following the second application, significant tooth whitening was achieved. To complete the treatment, the access cavity was restored using a composite resin and bonding system (GC Corporation, Tokyo, Japan) (Figure 2a, 2b).

The patient was followed up for 12 months, with a radiograph showing complete root formation and reduction in lesion size (Figure 1d).

## Discussion

Various materials, including calcium hydroxide, MTA, and Biodentine, are suitable for apexification procedures [18]. While calcium hydroxide requires a prolonged period for apical barrier formation, MTA and Biodentine offer enhanced sealing properties and shorter setting times, making them increasingly popular choices. In cases of immature permanent teeth with necrotic pulp or apical periodontitis, a modified apexification approach is often preferred over REPs. This method facilitates the placement of an intraarticular post or composite resin core in the cervical third of the root canal, thereby enhancing the tooth’s resistance to fracture. Both apexification and revascularization techniques can promote continued root maturation. Research indicates that MTA apexification, when combined with composite resin or post-restoration, yields favorable long-term outcomes [19]. A clinical study with an average follow-up of 8.29 years reported zero tooth losses due to root fractures among immature teeth treated with MTA apexification [20].

In addition to facilitating cementum production and reparative dentin formation, MTA maintains the integrity of the pulp and is well tolerated by periradicular and periapical tissues [21,22]. As MTA expands during setting, it exhibits excellent sealing ability, particularly in a moist environment [23]. The recommended thickness of MTA for optimal sealing is 4 mm [24]. When set MTA is exposed to water, it releases calcium hydroxide, which possesses cementogenesis-inducing properties [25].

MTA can be placed using pluggers, guns, carriers, or ultrasonic condensation techniques. One study found that MTA demonstrates better adaptation with fewer voids when hand condensation is used compared to the ultrasonic method [26]. Conversely, another study reported a significantly better seal with the ultrasonic method. In our procedure, we utilized a plugger to compact the MTA effectively against the PRF barrier, as condensation pressure may also influence strength [27].

Discoloration resulting from trauma to a developing tooth can significantly impact young individuals due to aesthetic concerns. Noninvasive conservative procedures, such as bleaching, are generally preferable to invasive options like veneers or crowns [28]. Therefore, we employed a non-vital bleaching procedure, specifically the walking bleach method, after modified apexification. This approach is both cost-effective for the patient and can be completed quickly [29]. The patient expressed high satisfaction with the treatment due to the significant improvement in tooth shade.

## Conclusions

Based on our case report, we conclude that modified apexification followed by non-vital bleaching can be considered an effective choice for discolored trauma-affected teeth with incomplete root formation. More clinical trials are needed to ascertain this.
